# Sustained cardiac remodeling after a short-term very low calorie diet in type 2 diabetes mellitus

**DOI:** 10.1186/1532-429X-13-S1-P328

**Published:** 2011-02-02

**Authors:** Jacqueline Jonker, Marieke Snel, Sebastiaan Hammer, Rutger W van der Meer, Ingrid M Jazet, Hanno Pijl, A Edo Meinders, Johannes A Romijn, Johannes WA Smit, Albert de Roos, Hildo J Lamb

**Affiliations:** 1 LUMC, Leiden, Netherlands

## Introduction

A very low calorie diet (VLCD) in patients with type 2 diabetes mellitus (T2DM) results in cardiac remodeling and improved diastolic function. It is unknown how long these effects sustain after reintroduction of a regular diet.

## Purpose

To examine the long-term effects of initial weight loss by a VLCD on cardiac dimensions and function in T2DM patients.

## Methods

Fourteen patients with insulin-dependent T2DM (mean±SEM: age 53±2 years; BMI 35±1 kg/m^2^) were treated by a VLCD (450 kcal/day) during 16 weeks. Cardiac function was measured by magnetic resonance imaging before and after the 16-week VLCD and again after 14 months of follow-up on a regular diet.

## Results

Body mass index decreased from 35±1 kg/m^2^ to 28±1 kg/m^2^ after the VLCD and increased again to 32±1 kg/m^2^ at 18 months (both P<0.05 vs. baseline). Left ventricular (LV) mass and LV mass/LV end-diastolic volume ratio decreased after the 16-week VLCD ((119±8 to 102±7grams; 0.67±0.03 to 0.59±0.03 respectively (both P<0.05)) and remained decreased after 14 months of follow-up (respectively 109±9 grams; 0.56±0.03, both P<0.05 vs. baseline) The improvement in LV diastolic function, measured by the early (E) and atrial (A) diastolic filling phase ratio after the 16-week VLCD, was sustained after 14 months of follow-up (E/A ratio: 0.96±0.07 (baseline); 1.12±0.06 (after VLCD); 1.06±0.07 (18 months, P<0.05 vs. baseline)).

## Conclusions

Weight reduction by a 16-week VLCD in T2DM patients results in sustained cardiac remodeling and improved diastolic function after 14 months of follow-up, despite weight regain on a regular diet.

